# Vestibular Effects of a 7 Tesla MRI Examination Compared to 1.5 T and 0 T in Healthy Volunteers

**DOI:** 10.1371/journal.pone.0092104

**Published:** 2014-03-21

**Authors:** Jens M. Theysohn, Oliver Kraff, Kristina Eilers, Dorian Andrade, Marcus Gerwig, Dagmar Timmann, Franz Schmitt, Mark E. Ladd, Susanne C. Ladd, Andreas K. Bitz

**Affiliations:** 1 Erwin L. Hahn Institute for Magnetic Resonance Imaging, University Duisburg-Essen, Essen, Germany; 2 Institute for Diagnostic and Interventional Radiology and Neuroradiology, University Hospital Essen, Essen, Germany; 3 Department of Neurology, University Hospital Essen, Essen, Germany; 4 Siemens Healthcare Sector, Erlangen, Germany; 5 Division of Medical Physics in Radiology (E020), German Cancer Research Center (DKFZ), Heidelberg, Germany; University of Manchester, United Kingdom

## Abstract

Ultra-high-field MRI (7 Tesla (T) and above) elicits more temporary side-effects compared to 1.5 T and 3 T, e.g. dizziness or “postural instability” even after exiting the scanner. The current study aims to assess quantitatively vestibular performance before and after exposure to different MRI scenarios at 7 T, 1.5 T and 0 T. Sway path and body axis rotation (Unterberger's stepping test) were quantitatively recorded in a total of 46 volunteers before, 2 minutes after, and 15 minutes after different exposure scenarios: 7 T head MRI (n = 27), 7 T no RF (n = 22), 7 T only B_0_ (n = 20), 7 T in & out B_0_ (n = 20), 1.5 T no RF (n = 20), 0 T (n = 15). All exposure scenarios lasted 30 minutes except for brief one minute exposure in 7 T in & out B_0_. Both measures were documented utilizing a 3D ultrasound system. During sway path evaluation, the experiment was repeated with eyes both open and closed. Sway paths for all long-lasting 7 T scenarios (normal, no RF, only B_0_) with eyes closed were significantly prolonged 2 minutes after exiting the scanner, normalizing after 15 minutes. Brief exposure to 7 T B_0_ or 30 minutes exposure to 1.5 T or 0 T did not show significant changes. End positions after Unterberger's stepping test were significantly changed counter-clockwise after all 7 T scenarios, including the brief in & out B_0_ exposure. Shorter exposure resulted in a smaller alteration angle. In contrast to sway path, reversal of changes in body axis rotation was incomplete after 15 minutes. 1.5 T caused no rotational changes. The results show that exposure to the 7 Tesla static magnetic field causes only a temporary dysfunction or “over-compensation” of the vestibular system not measurable at 1.5 or 0 Tesla. Radiofrequency fields, gradient switching, and orthostatic dysregulation do not seem to play a role.

## Introduction

Since its introduction, magnetic resonance imaging (MRI) has been known as a safe diagnostic imaging procedure. It has become a standard diagnostic tool from 0.6 T systems in the late 1970s to 1.5 T scanners in the mid-1980s and clinical 3 T systems starting in 2000/2001 [1]. Contemporaneous with the establishment of MRI systems at 3 T and above, the subjective intensification of the transient side-effects “vertigo” and “dizziness” has been accepted as an effect prompted by disturbance of the vestibular organ that becomes stronger with higher field strength [2]. The first human 8 T scanner was inaugurated for research purposes in 1998 [3]. By early 2014, approximately 45 ultra-high field (UHF) scanners for human imaging at or above 7 T will be in operation worldwide, with roughly 10% operating at 9.4 T and solitary systems at 10.5 T (whole-body) and 11.7 T (head).

Lasting side-effects after over 100 million MRI examinations worldwide, including multiple thousand examinations at 4 T to 9.4 T, have not been reported so far. Nevertheless, the application of these ultra-high-field MRI systems in humans has led to a raised consciousness regarding safety effects on volunteers, patients, operators, and service personnel [4]. Temporary side-effects including vertigo have been evaluated in the context of the subjective acceptance of 7 T and 9.4 T MRIs following its broader utilization [5], [6], [7]. Many short-term effects related to exposure to time-varying or static main magnetic fields have been described with non-serious consequences for humans [8]. Most often they have been discussed in the context of high field [9], [10], [11], [12], [13], [14], [15], [16], but are not specific to ultra-high-field systems. Yet, officially, only field strengths up to 8 T (FDA, United States), and up to 4 T (IEC, Europe), respectively, are considered “without significant risk” (for humans older than 1 month) [17], [18], and so far, scanners operating at field strengths beyond 4 T are only used in research. While the perception of physiological side-effects was not new, lower field MRI machines (1.5 T and below) caused such effects only in a very small number of more sensitive individuals. Only over the last 10 years has the interest in further understanding these effects become acute, leading to the publication of numerous papers on the interaction of magnetic fields with the vestibular organ including possible explanations of the underlying physiological mechanisms [4], [9], [10], [12], [15], [16], [19], [20], [21], [22].

The static magnetic field B_0_ interacts with the human body at the molecular, cellular, tissue, and organ level. Looking at the vestibular organ in particular, different mechanisms have been proposed to play a role in the origin of illusory sensations: Glover et al. (2007) hypothesized as possible causes for vertigo susceptibility differences between vestibular organs and surrounding fluid or induced currents acting on hair cells could be held responsible. Roberts et al. (2011) excluded susceptibility and direct stimulation of hair cells as a possible cause, and identified the Lorentz force inside the lateral semicircular canal as the best explanation for excitation of sensory organs; currents through ion transport between endolymph fluid and hair cells would interact with the static magnetic field and generate a low Lorentz force moving the endolymph and causing a subtle displacement of the cupulas, thereby inducing nystagmus and the sensation of vertigo. The authors elaborate on a possible adaptation to persistent vestibular stimulation in which the sense of rotation stops after introduction into the bore, while nystagmus persists, reaches a plateau after 10 minutes, and decays a few minutes after exit from the scanner. This mechanism was examined in more detail by Mian et al. [23]. Decreasing sense of rotation after reaching the isocenter has been a general perception for most healthy subjects and patients, and many authors thus concluded that the effects must be associated with movement through the static magnetic field or magnetic field gradient rather than a dependency on the level of the magnetic field itself. The adaptation hypothesis, however, enables a mechanistic explanation only dependent on the magnitude and direction of the magnetic field.

Thormann et al. (2013) showed that diphenhydramine, a medication frequently used to prevent motion sickness, can reduce vertigo after a one minute exposure, but without showing data on nystagmus. Currents may also occur in the stationary situation due to flowing blood [19], [24], but larger vessels are too far away to have an effect on the inner ear. Electromagnetic induction through movement of the entire body into the bore, i.e. from outside the scanner room into the center of the scanner (maximum field strength), is a well-known phenomenon, with induced currents inside the body depending on dB_0_/dt. Due to stronger field gradients in the stray field of high-field magnets, movement through this spatially variable field might be the most important difference compared to lower-field magnets regarding side-effects [8]. Yet the stray fields of passively shielded 7 T magnets and actively shielded 3 T magnets are not too different regarding the magnetic field gradient dB_0_/dz. Vertigo after stray field exposure to a 7 T scanner has been studied by van Nierop et al. (2013), who evaluated the body sway of subjects after sitting in front of a 7 T MRI for one hour and in the end moving their heads for 16 seconds. Sway path length, sway area, and sway velocity were significantly higher with higher static magnetic field, and all three measures proved to be highly correlated.

The gradient system produces weak magnetic fields which superimpose on the static magnetic field. In 7 T systems, gradient performance is similar or identical with modern 1.5 T and 3 T systems, as all systems are made to observe the IEC 60601-2-33 guidelines [18] for switching gradients. Thus, effects associated with gradients (e.g. peripheral nerve stimulation) are not expected to differ at higher fields.

RF pulses for spin excitation will deposit energy in the tissue and generate heat. The IEC and FDA limits for the maximum rate of energy deposition in the body tissue and for the maximum tissue temperature do not depend on the frequency and, thus, conditions at 7 T are similar to lower-field systems. There is no evidence that RF pulses play a role in the induction of vertigo and related side effects.

Thus, it is expected that the main difference between a 7 T system and lower-field systems regarding side-effects involving the vestibular organ will be related to effects induced by the static magnetic field. Of course one might also consider a collective effect on the organism as responsible, but we are not aware of any theories proposing this.

Subjects who undergo an ultra-high-field MR examination often report a subjective sensation of vertigo during introduction into the bore. But in our experience some also report a form of “postural instability” persisting after the examination, even outside the scanner room, and in individual cases even reported the following day. To obtain information regarding the duration of such effects and the responsible physical electromagnetic fields, the current study aims at quantitatively assessing the vestibular performance by measuring a) postural instability by means of body sway and b) rotational divergences by means of Unterberger's stepping test, both before and after exposure to different MRI scenarios at 7 T, 1.5 T, and 0 T. Scenarios were included both with and without exposure to RF and gradient magnetic fields.

## Materials and Methods

The University Hospital Essen ethics committee authorized the examinations as part of fundamental single center research on high-field MR (permit no. 06-3117). Written consent was obtained from all subjects.

### Study layout

In this two-phase study, forty-six neurologically healthy volunteers (37 m, 19 f, mean age 32.8 y) underwent tests before, 2 minutes after, and 15 minutes after six different brain MR exposure scenarios at three different field-strengths (7 T, 1.5 T, and 0 T). In four scenarios a 7 T MRI was utilized, while one scenario involved a 1.5 T MRI and one was done without MR exposure. They differed by selectively turning off radiofrequency excitation (no RF), deactivating the gradients (no GR), or by minimizing exposure duration to the static field by moving subjects into the bore and directly out again without a longer stationary dwell time inside the magnet (in & out). In phase one (n = 26, 17 m, 9 f, mean age 24.3 y) three exposure scenarios (‘7 T’, ‘7 T no RF’ and ‘0 T’) were evaluated using Romberg's test (body sway), while in phase two (n = 20, 10 m, 10 f, mean age 43.8 y, n = 10 age <30 y, n = 10 age >50 y) volunteers were exposed to one identical (‘7 T no RF’) and three new scenarios (‘7 T no RF & no GR’, ‘7 T in & out’ and ‘1.5 T no RF’) and Unterberger's stepping test was added to the measurements. The exposure for all scenarios lasted 30 minutes except for ‘7 T in & out’, which took about one minute. No volunteer took part in both phases. Only one exposure scenario was tested on any given day.

#### Phase one

During the first phase, 26 volunteers were exposed to a normal 7 T head MRI examination (‘7 T’). Thirteen of these took part in an additional exposure scenario with deactivated RF transmission by manually setting transmit amplitude to zero (‘7 T no RF’), resulting in exposure only to the 7 T static magnetic field and the fields of the gradient coils. Most volunteers have had previous MRI experience and were blinded to RF deactivation. Furthermore, 16 of the volunteers were included in a control study without exposure to the physical fields produced by the MR system (‘0T’); instead, they rested on a gurney in a dark and quiet room for 30 minutes. Results of the control group were used to exclude physiological effects related to orthostatic regulation.

#### Phase two

In addition to one setting from phase one (‘7 T no RF’), twenty additional volunteers without previous MRI experience were newly exposed to the 7 T static magnetic field only (‘7 T no RF & no GR’) and were tested after movement into the magnet with immediate removal (‘7 T in & out’). Furthermore, an analogous ‘1.5 T no RF’ situation was evaluated. The order of the exposure scenarios was randomized in this phase, and Unterberger's stepping test was added after Romberg's test. Unterberger's stepping test was chosen based on preliminary tests performed before starting the second phase that indicated its usefulness. Other additional motor tests that were evaluated did not seem useful (arm extension test, 9-hole peg test, line walk). Volunteers in two age groups with an equal distribution of genders were included; the age groups were younger than 30 years (n = 10) and older than 50 years (n = 10).

**Figure 1:**
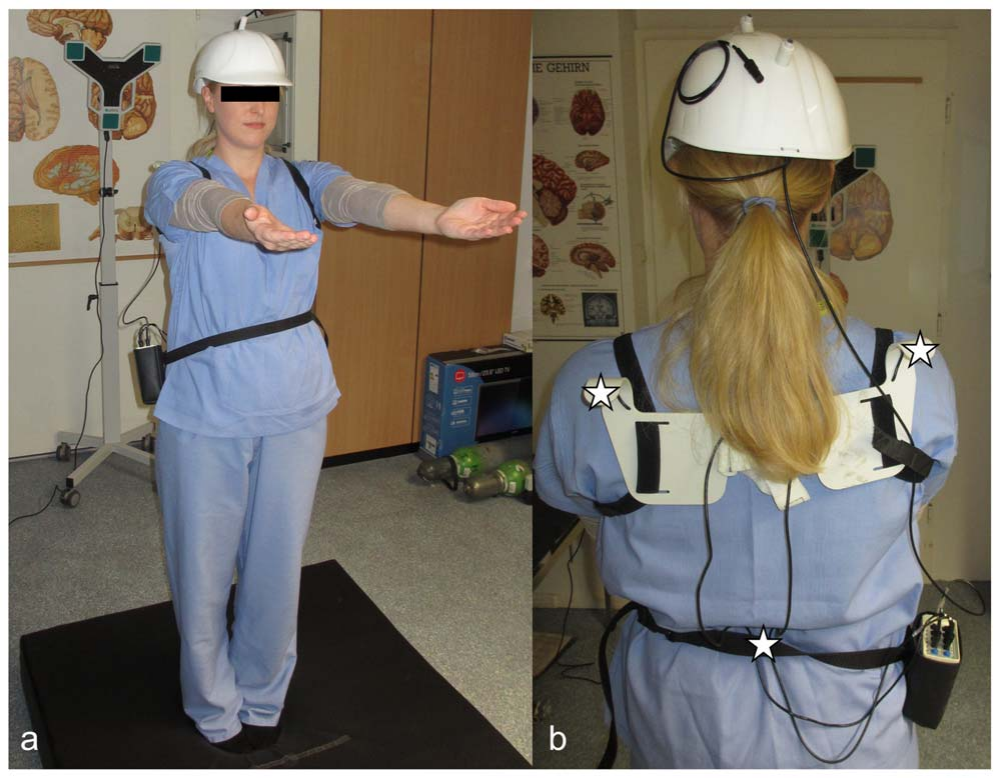
Test arrangement. (a) Body posture during Romberg's test for sway path evaluation with the feet close together, arms straight forward, palms facing up, and eyes closed (top). 20-cm-thick foam cushion used to minimize proprioceptive feedback during sway path evaluation (bottom). (b) Three ultrasound transmitters (*) positioned on the two shoulders and the lumbar spine were used for data generation.

**Figure 2:**
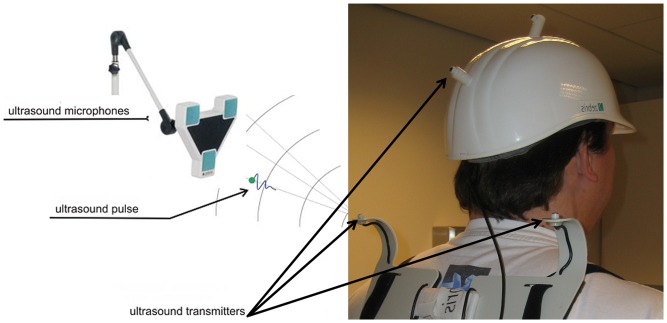
Posture analysis system. Multiple ultrasound transmitters were positioned on the body during Romberg's and Unterberger's tests; a real-time ultrasound measuring system (Zebris Medical Systems, Isny, Germany) was used to record the 3D positions of these transmitters.

Romberg's test was performed for 30 seconds while standing on a 20-cm-thick foam cushion with the feet close together and with open or closed eyes while wearing ear plugs. Romberg's test was defined as standing straight with arms forward and parallel and palms facing up (Figure 1a). The foam cushion (Figure 1b) was used in order to minimize the proprioceptive feedback from the lower extremities usually used to maintain balance. Ear plugs were used to dampen surrounding noise and reduce the possibility to orient using acoustic cues. Stability of stance is usually based on proprioceptive, vestibular, and visual information. When standing on the cushion with eyes closed, stance stability is heavily dependent on vestibular system function [25]. Unterberger's stepping test was recorded for 30 seconds while holding the arms forward and parallel with the palms facing up and stepping on the spot with eyes closed. Analysis of the body motion for both tests was performed by a real-time ultrasound measuring system (Zebris Medical Systems, Isny, Germany) capable of recording the 3D positions of transmitters fixated to the body (Figure 2). After initial calibration of the system, the travelled distance of the lumbar transmitter (sway path) was recorded during Romberg's test, and the rotation of the body axis as given by the line through the transmitters on each shoulder was recorded during Unterberger's stepping test, in each case for 30 seconds. A positive rotation was defined as clockwise rotation when looking down on the head of the volunteer.

### MR exposure

The MR exposure lasted 30 minutes and was performed with a whole-body 7 T MRI (Magnetom 7T, Siemens Healthcare, Erlangen, Germany) equipped with a gradient system capable of 45 mT/m maximum amplitude and a slew rate of 220 mT/m/ms. The polarity of the magnetic field was directed from the head of the patient table to the foot of the patient table. RF transmission was selectively performed using an eight-channel transmit/receive head coil (Rapid Biomed, Wurzburg, Germany). The 7 T scanner is not equipped with a built-in transmit body coil. The subjects were introduced into the bore in the ‘head-first supine’ position after being placed in the head coil. The motorized patient table was moved into the bore following a drive profile with reduction to slow speed (3.8 cm/s) during strongest B_0_ x dB_0_/dz to reduce the likelihood of inducing sizeable currents. Volunteers were advised to close their eyes during movement and the stationary phase inside the MRI, but were free to open them if they felt comfortable doing so. In those cases where they had their eyes open, visual feedback should have lessened the vestibular disturbance, as is known for patients with vestibular disorders [26]. Lights inside the magnet were dimmed. For the 1.5 T scenario a clinical system was utilized (Avanto, Siemens Healthcare, Erlangen, Germany) with identical bore diameter (60 cm) and similar gradient system. Although no RF was used at 1.5 T, the volunteers were positioned inside a standard receive-only head coil. Where applicable, a fixed protocol of sequences lasting 28:42 min. plus initial shimming was used at both field strengths: magnetization-prepared rapid acquisition gradient echo (6:27 min.), proton-density/T2 turbo spin-echo (4:13 min.), susceptibility-weighted imaging (8:04 min.), fluid-attenuated-inversion-recovery (7:16 min.), and T2* (2:42 min.) were run in the given order.

### Data analysis

Data were evaluated to determine the sway path length of the lumbar spine transmitter over 30 seconds and the rotation angle of the shoulder axis after 30 seconds. For Romberg's test data were acquired both with eyes open and closed. For Unterberger's test, the pre-exposure rotation was used as reference for the subsequent measurements and differences in rotation angle were analyzed. Statistical analysis was performed for the three different observations and the individual exposure scenarios using one-way ANOVA for repeated measurements and post-hoc Bonferroni correction. For subgroup analysis in phase 2 an additional gender by age two-way ANOVA of delta-sway path and delta-step test (pre and 2′) was performed.

## Results

Tables S1 and S2 give an overview of all results (phase one and two, Romberg's test and Unterberger's stepping test) including standard deviation and significance.

### Phase one

**Figure 3:**
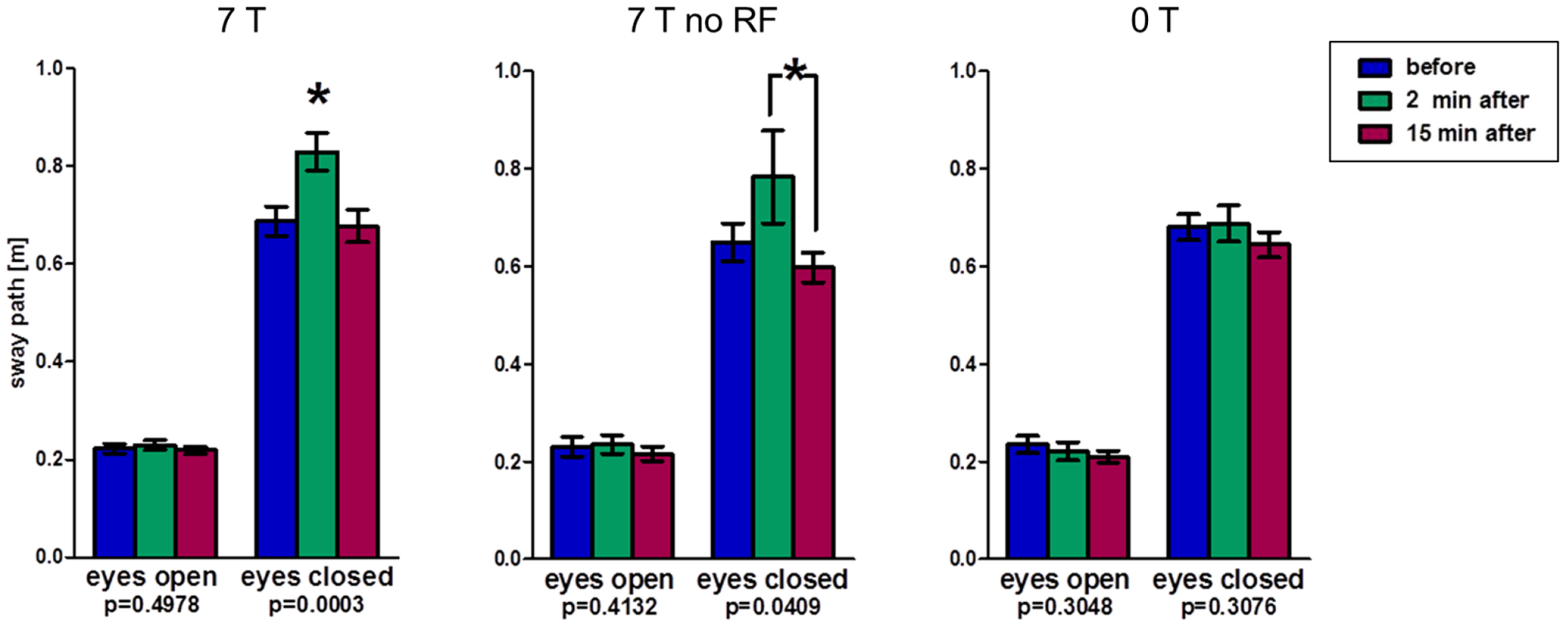
Romberg's test results (phase 1) indicating postural in-/stability by means of sway path length of the lumbar spine. Results of the Romberg's test of phase 1 displaying sway path lengths of the lumbar spine as an indicator of postural stability before, 2 minutes after, and 15 minutes after three different exposure scenarios. p-values of one-way ANOVA test for repeated measurements and (*) post-hoc Bonferroni with p<0.05.

In Figure 3 the sway path length of the lumbar spine, or trunk, respectively, is shown as a measure for postural stability in the volunteers. Results for tests with eyes open show no significant differences in postural stability between experiments before and after exposure (all mean sway paths between 0.21 m and 0.24 m); these sway paths were roughly one third compared to those recorded with the eyes closed before exposure (range: 0.65 m–0.69 m). For eyes closed, the sway path showed significant changes between the different time points. After exposure of 26 volunteers to a normal 7 T MRI (‘7 T’), the mean sway path was significantly increased 2 min. after the MRI examination (0.83 m vs. 0.69 m), indicating a postural instability. This instability normalized after 15 min. (0.68 m; ANOVA p<0.01). An analogous significant increase in sway path length (0.78 m vs. 0.65 m) was also apparent directly after 7 T exposure without RF (n = 13), with again a significant drop between 2 min. and 15 min. post exposure (0.60 m; ANOVA p = 0.04). Sway paths for the control group (n = 16) without any magnetic field exposure showed no significant changes after resting for 30 min. (0.68 m, 0.69 m, and 0.64 m).

### Phase two

**Figure 4:**
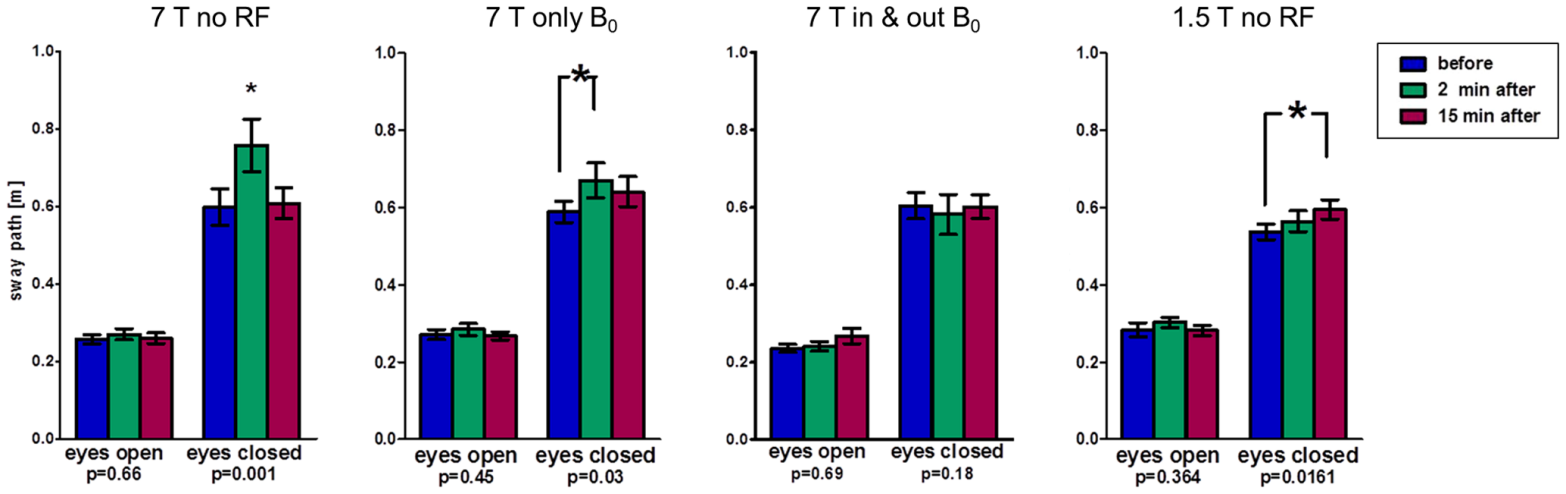
Romberg's test results (phase 2) indicating postural in-/stability by means of sway path length of the lumbar spine. Results of the Romberg's test of phase 2 displaying sway path lengths of the lumbar spine as an indicator of postural stability before, 2 minutes after, and 15 minutes after four different exposure scenarios. p-values of one-way ANOVA test for repeated measurements and (*) post-hoc Bonferroni with p<0.05.

**Figure 5:**
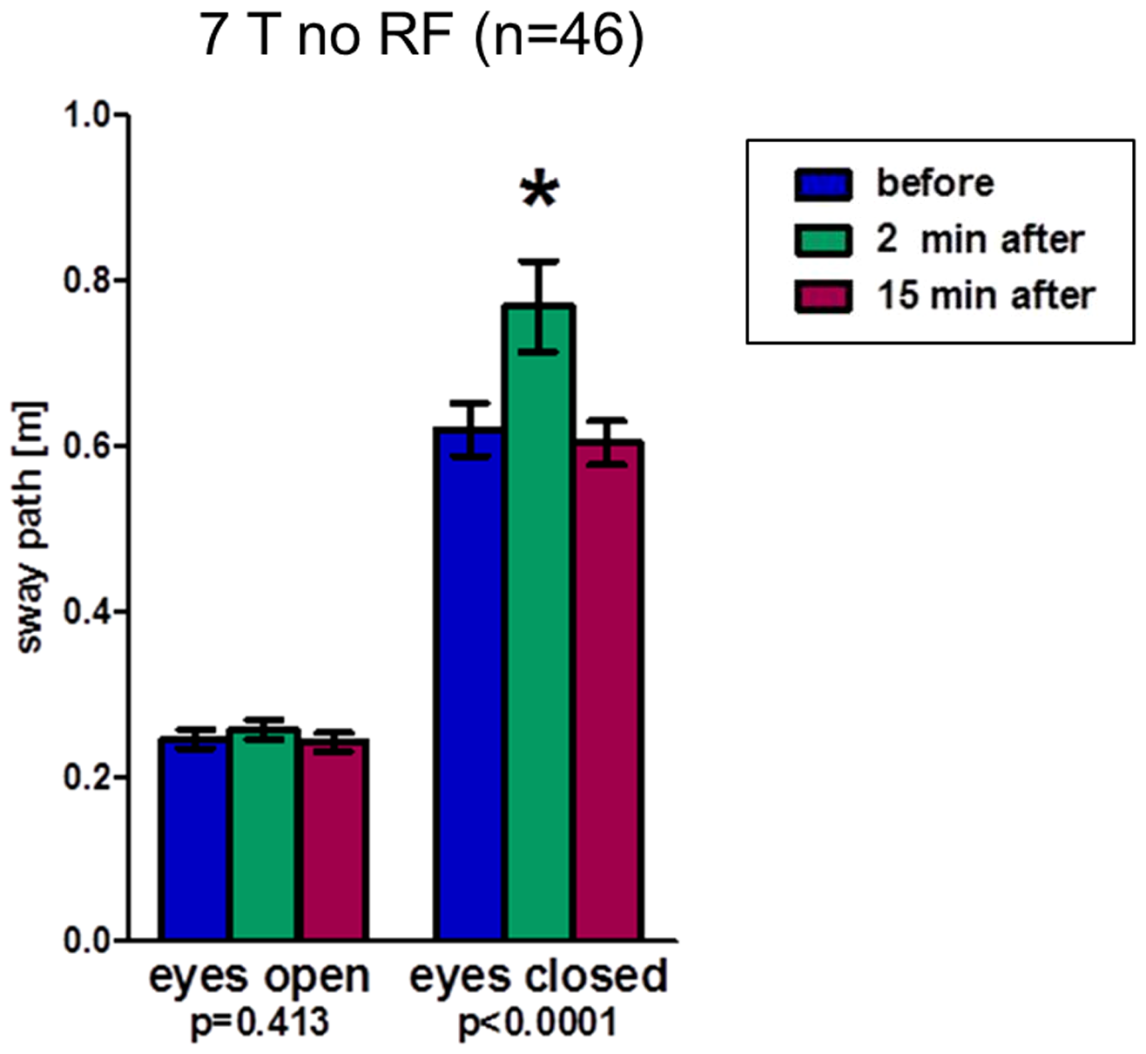
Romberg's test results (phases 1+2) indicating postural in-/stability by means of sway path length of the lumbar spine. Results of the Romberg's test of all subjects of phase 1 and 2 (n = 46) displaying sway path lengths of the lumbar spine as an indicator of postural stability before, 2 minutes after, and 15 minutes after ‘7T no RF’ exposure. p-values of one-way ANOVA test for repeated measurements and (*) post-hoc Bonferroni with p<0.05.

A new group of 20 volunteers was then exposed to three different scenarios at 7 T and one scenario at 1.5 T (Figure 4). Again, measurements with open eyes resulted in no significant changes in mean sway path length between time points and scenarios (range: 0.23 m–0.30 m). Results for closed eyes were as follows: ‘7 T no RF’ resulted in significant temporary increase after exposure as observed in phase one (mean 0.60 m, 0.76 m, 0.61 m; ANOVA p = 0.001); combined results of both phases including 46 volunteers support these data (0.62 m, 0.77 m, 0.60 m; ANOVA p<0.01; Figure 5). While 30 min. exposure to the static magnetic field only results in significant increase in sway path length 2 min. after (0.59 m, 0.67 m; ANOVA p = 0.03), it normalizes after a period of 15 min. (0.64 m; p>0.05). A complete, but brief, introduction into the bore (‘7 T in & out’) is not associated with varying sway paths (0.60 m, 0.58 m, 0.60 m; ANOVA p = 0.18). Finally, ‘1.5T no RF’ produced significant results, but with a steady moderate increase of sway path length even 15 min. after exposure (0.54 m, 0.56 m, 0.60 m; ANOVA p = 0.02).

**Figure 6:**
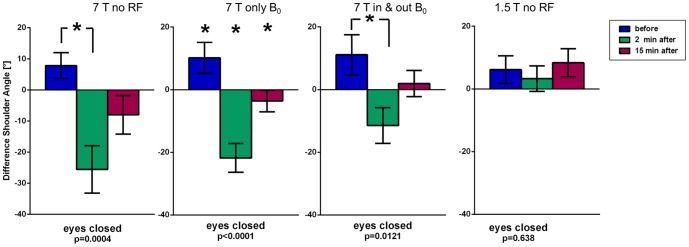
Unterberger's test results (phase 2) indicating rotational behavior of body. Results of the Unterberger's stepping test of phase 2 displaying body axis rotation before, 2 minutes after, and 15 minutes after four different exposure scenarios. p-values of one-way ANOVA test for repeated measurements and (*) post-hoc Bonferroni with p<0.05.

The Unterberger's test (Figure 6) resulted in an absolute mean shoulder rotation between 5° and 11° clockwise (looking down at the head) before exposure (control measurement). Significant effects (p<0.05) occurred for rotations 2 min. after ‘7 T no RF & no GR’ and ‘7 T no RF’, with a mean of −40° and −38° (counter-clockwise) relative to the control measurements, respectively. Notably, the ‘7 T in & out’ scenario also showed a counter-clockwise rotation at 2 min. after exposure (−23° relative to control measurement). Only rotation 2 min. after 1.5 T exposure was unchanged (−3° counter-clockwise relative to pre-exposure recording). All significant changes showed an incomplete normalization after 15 minutes to −23° (‘7 T no RF & no GR’), −18° (‘7 T no RF’), and −10° (‘7 T in & out’) relative to the control at 7 T.

**Figure 7:**
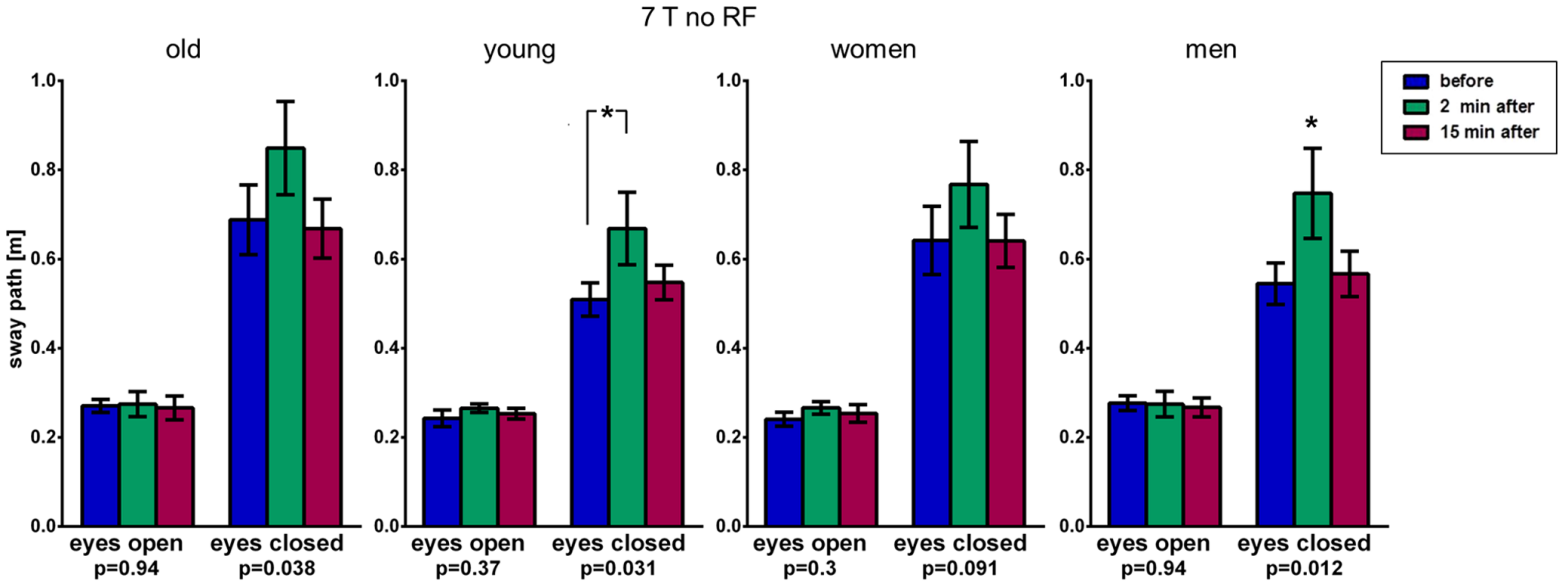
Subgroup analysis of Romberg's test results (phase 2) indicating postural in-/stability by means of sway path length of the lumbar spine. Subgroup analysis of results of the Romberg's test of phase 2 (Figure 4) comparing young (<30 y) vs. old (>50 y) and men vs. women. The older age group seems to generate slightly longer sway paths after 7 T exposure for 30 minutes. See also Figures S1–3. P-values of one-way ANOVA test for repeated measurements and (*) post-hoc Bonferroni with p<0.05.

Analysis of smaller subgroups (n = 10 each) regarding age (<30 y/>50 y) and gender (male/female) revealed some apparent trends between the age groups that are visible when looking at the figures, but are statistically less reliable due to the sample size (one-way ANOVA: Figures 7, S1–S3). Two-way ANOVA did only generate two borderline significances which are mentioned in parentheses. The older age group seems to generate slightly longer sway paths with closed eyes for ‘7 T no RF’ and ‘7 T no RF & no GR’ (two-way ANOVA p = 0.066) before and after exposure. At the same time, changes in rotation 2 min. after all 7 T exposures in the Unterberger's stepping test are about 10° smaller than in the younger group, including the two aforementioned scenarios and ‘7 T in & out’). While gender comparison revealed a stronger sway path increase in men for ‘7 T no RF’ (one-way ANOVA: Figure 7; two-way ANOVA, p = 0.054), men showed no changes after ‘7T only B_0_’ with women having a borderline significant (p = 0.051) sway path increase (Figure S1). The ‘1.5 T no RF’ scenario did not unveil any dependency (Figure S3).

## Discussion

Our study provides further data regarding temporary vestibular changes through high-field MRI that has not been acquired before and can be well integrated into the discussion of underlying mechanisms. Our data support the general understanding that the gradient system and RF excitation do not play a role in the generation of vertigo, since turning off both MR components individually did not diminish temporarily measurable postural instability and rotational mis-perception. At the same time the data strongly support that changes are attributable to the vestibular system, since all effects were suppressed by performing the tasks with eyes open, proprioceptive feedback was minimized by standing on a foam cushion (during Romberg's test) and physiological effects due to orthostatic regulation after returning to the upright position could also be excluded. We propose that the measurable effects are due to compensatory adaptation processes inside the vestibular organ, re-adapting to the normal state after terminating the exposure.

Glover et al. (2007) elaborated on three theories of disturbance of the vestibular organ [10]: changed firing rates of hair cells through induced currents, magneto-hydrodynamics, and subtle forces induced by the static magnetic field due to susceptibility differences of soft tissues. The authors concluded that the first effect could explain vertigo during movement through a changing field and that the latter mechanism could explain effects during stationary positioning within a static magnetic field through forces on the diamagnetic otolithic membrane and/or cupula. Our results capture only changes persistent after leaving the magnetic field and cannot differentiate between the causes. Our data, however, partially support the adaptation process of the vestibular organ suggested by Roberts et al. (2011) and Mian et al. (2013), since the effects on sway path were only detectable after an extended period in the 7 T field. Brief exposure as in the ‘7 T in & out’ scenario did not significantly alter sway path; shoulder rotation as captured by Unterberger's stepping test, on the other hand, was affected even after brief exposure. The measured effects of elongated sway path and changed rotational perception both persisted for a few minutes after leaving the magnetic field, with the effect on shoulder rotation still detectable even 15 min. after exposure, whereas the sway path returned to normal within this time. We cannot definitively conclude whether the cause of a directional turning sensation upon introduction into the bore is identical with the one inducing adaptation processes measurable after exit, but this appears likely. Since we propose the Unterberger's test to be more dependent on vestibular input than the Romberg's test, it is reasonable for it to show changes for a longer time.

Roberts et al. (2011) observed the appearance of a nystagmus in all healthy subjects when stationary in a magnetic field that was not visible in patients with no labyrinthine function [27]. Attributing this effect to the vestibulo-ocular reflex, the direction and velocity of the nystagmus were recorded. According to the explanation proposed by the authors, effects during introduction into the magnet causing vertigo (higher-threshold sensation) are stronger and die away after becoming stationary, but lower-threshold nystagmus persists. Similar to their results we show that effects on the vestibular system are stronger with higher fields and with longer exposure: our 7 T measurements show significant changes after 30-minute exposures, which are smaller or not measurable after very short (1 minute) exposure or at lower field strength (1.5 T). While they only descriptively indicate that the nystagmus decays a few minutes after exciting the bore, graphical data suggest this to be around 3–4 minutes; our study setup measuring two minutes after exit is compatible with this decay timeframe. Roberts et al. argued against susceptibility effects playing a role due to the dependency of nystagmus direction on field polarity. They further documented a dependency of nystagmus intensity on the orientation of the lateral canal inside the static field; no nystagmus was measurable in any subject for an individually definable head position (tilt of the chin up or down), but with a very variable range (−27°–+32°). This variability of receptivity can partially explain rather large standard deviations in some of our data points. Our subjects all had a similar and fixated head position inside the scanner due to the placement into the head coil in all scenarios. It would be interesting to investigate whether our data could be reproduced when exposing subjects only in their “no nystagmus” orientation.

While we analyzed postural body sway after an MRI examination, Van Nierop et al. (2013) only exposed subjects to the stray field, combining static exposure for one hour followed by additional head movement for 16 seconds at different distances from the scanner [28]. Although their aim was different from our study, the authors could show high correlations between the body sway length, the area under the body sway curve, and the velocity of body movements; thus, our focus on the evaluation of body sway path length only seems reasonable.

Thormann et al. demonstrated that diphenhydramine, usually administered to prevent motion sickness, can reduce subjective vertigo after a one-minute exposure to 7 T [29]. It would be interesting to see if it could also modulate nystagmus, body sway path, and/or Unterberger's test performance.

In a recent study regarding healthy older adults, Davalos-Bichara and Agraval [30] documented the observation that older individuals often performed less well in vestibular tests. Although their age group (>70 y) was older than our subgroup (>50 y) it might explain why we experienced slightly prolonged sway paths before and after exposure compared to the younger (<30 y) subgroup. The lesser sway path increase of the young group in our “7 T no RF & no GR” subgroup (Figure S1) might be due to the small sample size (n = 10); since the “7 T no RF” subgroup (Figure 7) shows comparable increases after 2 minutes in both age groups a dependency on age seems less likely. Faraldo-Garcia et al. proposed vestibular information for postural control to decrease beginning with 50 years of age due to aging of the vestibular system [31] which could also explain our data. In the Unterberger's stepping test, on the other hand, temporary changes in rotation 2 min. after all 7 T exposures are about 10° smaller in the older subgroup than in the younger group; although we proposed older volunteers to have a lower vestibular performance, this might be due to a less responsive vestibular system in older subjects or possibly due to lower body activity and smaller steps during the test. Not finding any dependency on gender for any of the 7 T or 1.5 T data agrees with the literature [31].

Limitations of the presented study include the assumption that the increased body movement, as captured by the setup to measure postural instability, can be held equivalent to a vestibular dysfunction. Moreover, for better comparability the control group at 0T could have been placed into a mock scanner, which was not available. In addition, the population group was relatively small, but on the other hand, it was large enough generate statistically significant results in many comparisons. We were also not able to differentiate dependencies on magnet field direction, since all subjects were introduced head-first from the front of the magnet. Furthermore, it would be interesting to obtain similar data at 3 T in future work, since 3 T is the current upper limit of clinically available MRI systems.

The results show that exposure to magnetic and/or electromagnetic fields produced by a 7 Tesla MR system during examination of the head only temporarily causes a dysfunction or “over-compensation” of the vestibular system while returning to its normal state. This effect does not seem to be related to exposure to RF energy or gradient switching and seems only to be dependent on the duration and field strength of magnetic field exposure. The practical consequence of the vestibular disturbance detected in this study is unclear, and further studies will be needed to more comprehensively determine the implications of our findings on patients, volunteers, or workers exposed to high magnetic fields. It may be advisable for persons exposed to high static magnetic fields to temporarily avoid certain locomotor tasks dependent on vestibular input such as climbing ladders or operating motor vehicles. Unfortunately, our data do not provide sufficient guidance regarding these issues. They do, however, indicate that vestibular function largely returns to normal within 15 minutes, so that a suitable waiting time should be adequate to assure normal vestibular function.

## Supporting Information

Figure S1 Subgroup analysis (“7T only B_0_”) of Romberg's test results (phase 2) indicating postural in-/stability by means of sway path length of the lumbar spine. Subgroup analysis of results of the Romberg's test of phase 2 (Figure 4) comparing young (<30 y) vs. old (>50 y) and men vs. women. The older age group seems to generate slightly longer sway paths after 7 T exposure for 30 minutes. See also Figure 7. P-values of one-way ANOVA test for repeated measurements and (*) post-hoc Bonferroni with p<0.05.
(TIF)

Figure S2 Subgroup analysis (“7T in & out B_0_”) of Romberg's test results (phase 2) indicating postural in-/stability by means of sway path length of the lumbar spine. Subgroup analysis of results of the Romberg's test of phase 2 (Figure 4) comparing young (<30 y) vs. old (>50 y) and men vs. women. No subgroup shows significant changes after 7 T exposure for 1 minute. See also Figure 7. P-values of one-way ANOVA test for repeated measurements and (*) post-hoc Bonferroni with p<0.05.
(TIF)

Figure S3 Subgroup analysis (“1.5T no RF”) of Romberg's test results (phase 2) indicating postural in-/stability by means of sway path length of the lumbar spine. Subgroup analysis of results of the Romberg's test of phase 2 (Figure 4) comparing young (<30 y) vs. old (>50 y) and men vs. women. No subgroup shows significant changes after 1.5 T exposure for 30 minutes. See also Figure 7. P-values of one-way ANOVA test for repeated measurements and (*) post-hoc Bonferroni with p<0.05.
(TIF)

Table S1 Romberg's test results (phases one and two). Results of the Romberg's test show significant changes of sway path 2 minutes after most longer lasting 7 T exposure scenarios compared to the pre-exposure measurement (“pre/2”). Shorter 7 T exposure (“7 T in & out”) and 1.5 T exposure do not generate significant changes.
(DOCX)

Table S2 Unterberger stepping test results (phase 2). Results of the Unterberger's stepping test show significant changes of rotation 2 minutes after all 7 T exposure scenarios compared to the pre-exposure measurement (“pre/2”). Although only significant in the “7 T no RF & no GR” group (“2/15”), all changes are substantially but not completely reversed in the 15 minute measurement. Results after 1.5 T exposure do not change convincingly.
(DOCX)
