# Supplementary figures and images for: Vestibular Effects of a 7 Tesla MRI Examination Compared to 1.5 T and 0 T in Healthy Volunteers
Source: PLoS One. 2014 Mar 21;9(3):e92104. doi: 10.1371/journal.pone.0092104 (PMC3962400; doi:10.1371/journal.pone.0092104)

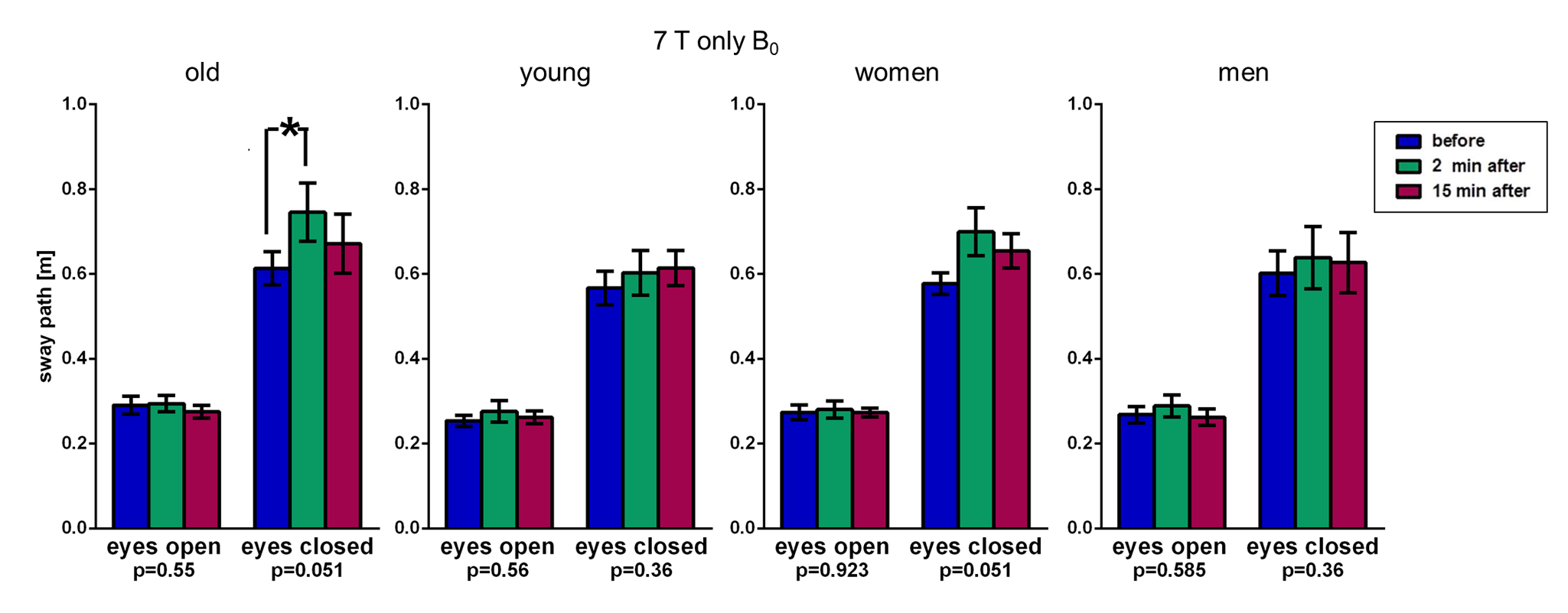

Supplement: Figure S1 — Subgroup analysis (“7T only B0”) of Romberg's test results (phase 2) indicating postural in-/stability by means of sway path length of the lumbar spine. Subgroup analysis of results of the Romberg's test of phase 2 (Figure 4) comparing young (<30 y) vs. old (>50 y) and men vs. women. The older age group seems to generate slightly longer sway paths after 7 T exposure for 30 minutes. See also Figure 7. P-values of one-way ANOVA test for repeated measurements and (*) post-hoc Bonferroni with p<0.05. (TIF) [file pone.0092104.s001.tif]

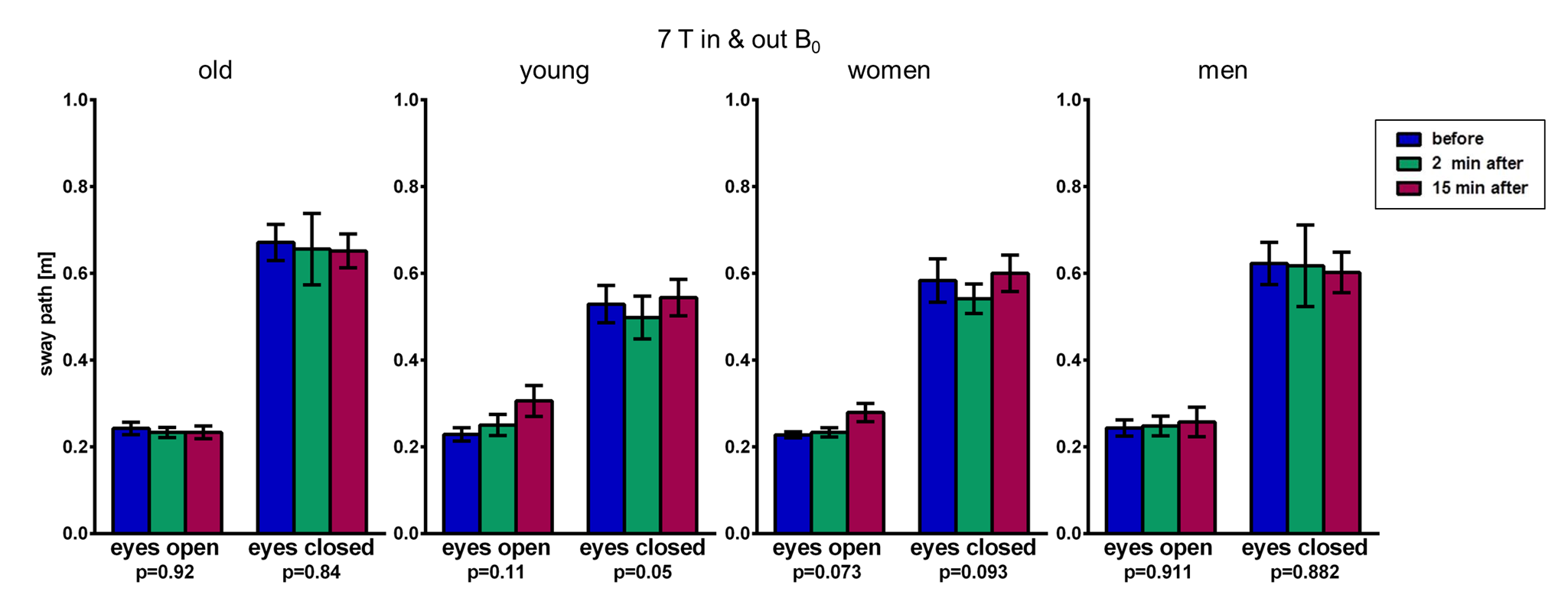

Supplement: Figure S2 — Subgroup analysis (“7T in & out B0”) of Romberg's test results (phase 2) indicating postural in-/stability by means of sway path length of the lumbar spine. Subgroup analysis of results of the Romberg's test of phase 2 (Figure 4) comparing young (<30 y) vs. old (>50 y) and men vs. women. No subgroup shows significant changes after 7 T exposure for 1 minute. See also Figure 7. P-values of one-way ANOVA test for repeated measurements and (*) post-hoc Bonferroni with p<0.05. (TIF) [file pone.0092104.s002.tif]

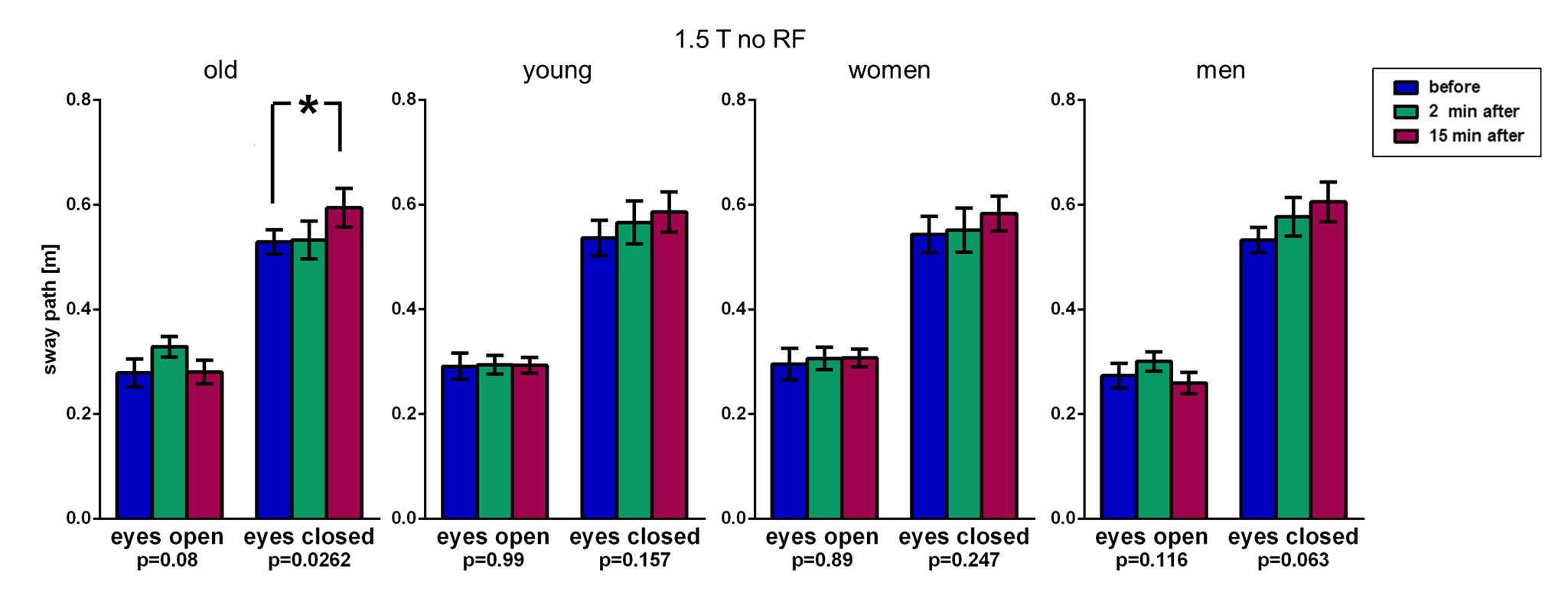

Supplement: Figure S3 — Subgroup analysis (“1.5T no RF”) of Romberg's test results (phase 2) indicating postural in-/stability by means of sway path length of the lumbar spine. Subgroup analysis of results of the Romberg's test of phase 2 (Figure 4) comparing young (<30 y) vs. old (>50 y) and men vs. women. No subgroup shows significant changes after 1.5 T exposure for 30 minutes. See also Figure 7. P-values of one-way ANOVA test for repeated measurements and (*) post-hoc Bonferroni with p<0.05. (TIF) [file pone.0092104.s003.tif]
