# Supplementary material for: Vestibular Effects of a 7 Tesla MRI Examination Compared to 1.5 T and 0 T in Healthy Volunteers
Source: PLoS One. 2014 Mar 21;9(3):e92104. doi: 10.1371/journal.pone.0092104 (PMC3962400; doi:10.1371/journal.pone.0092104)
Supplement: Table S1 — Romberg's test results (phases one and two). Results of the Romberg's test show significant changes of sway path 2 minutes after most longer lasting 7 T exposure scenarios compared to the pre-exposure measurement (“pre/2”). Shorter 7 T exposure (“7 T in & out”) and 1.5 T exposure do not generate significant changes. (DOCX) [file pone.0092104.s004.docx]

| \| **Table S1. Romberg's test results (phases one and two).** \| \| \| \| \| \| \| \| --- \| --- \| --- \| --- \| --- \| --- \| --- \| \| Phase \| Exposure group \| Eye state \| Time point \| Sway path [m] \| \| p < 0.05 \| \|  \|  \|  \|  \| Mean \| SD \|  \| \| Phase one \| 7 T (n = 26) \| open eyes \| pre \| 0.22 \| 0.05 \| - \| \|  \|  \|  \| 2 Min. \| 0.23 \| 0.05 \|  \| \|  \|  \|  \| 15 Min. \| 0.22 \| 0.04 \|  \| \|  \|  \| closed eyes \| pre \| 0.69 \| 0.15 \| pre/2, 2/15 \| \|  \|  \|  \| 2 Min. \| 0.83 \| 0.20 \|  \| \|  \|  \|  \| 15 Min. \| 0.68 \| 0.17 \|  \| \|  \| 7 T no RF (n = 13) \| open eyes \| pre \| 0.23 \| 0.07 \| - \| \|  \|  \|  \| 2 Min. \| 0.24 \| 0.07 \|  \| \|  \|  \|  \| 15 Min. \| 0.22 \| 0.05 \|  \| \|  \|  \| closed eyes \| pre \| 0.65 \| 0.14 \| 2/15 \| \|  \|  \|  \| 2 Min. \| 0.78 \| 0.34 \|  \| \|  \|  \|  \| 15 Min. \| 0.60 \| 0.11 \|  \| \|  \| 0 T (n = 16) \| open eyes \| pre \| 0.24 \| 0.07 \| - \| \|  \|  \|  \| 2 Min. \| 0.22 \| 0.07 \|  \| \|  \|  \|  \| 15 Min. \| 0.21 \| 0.05 \|  \| \|  \|  \| closed eyes \| pre \| 0.68 \| 0.11 \| - \| \|  \|  \|  \| 2 Min. \| 0.69 \| 0.15 \|  \| \|  \|  \|  \| 15 Min. \| 0.64 \| 0.10 \|  \| \| Phase two \| 7 T no RF (n = 18) \| open eyes \| pre \| 0.26 \| 0.05 \| - \| \|  \|  \|  \| 2 Min. \| 0.27 \| 0.06 \|  \| \|  \|  \|  \| 15 Min. \| 0.26 \| 0.06 \|  \| \|  \|  \| closed eyes \| pre \| 0.60 \| 0.20 \| pre/2, 2/15 \| \|  \|  \|  \| 2 Min. \| 0.76 \| 0.29 \|  \| \|  \|  \|  \| 15 Min. \| 0.61 \| 0.17 \|  \| \|  \| 7 T no RF & no GR (n = 19) \| open eyes \| pre \| 0.27 \| 0.06 \| - \| \|  \|  \|  \| 2 Min. \| 0.28 \| 0.07 \|  \| \|  \|  \|  \| 15 Min. \| 0.27 \| 0.40 \|  \| \|  \|  \| closed eyes \| pre \| 0.59 \| 0.12 \| pre/2 \| \|  \|  \|  \| 2 Min. \| 0.67 \| 0.20 \|  \| \|  \|  \|  \| 15 Min. \| 0.64 \| 0.17 \|  \| \|  \| 7 T in & out (n = 17) \| open eyes \| pre \| 0.23 \| 0.04 \| - \| \|  \|  \|  \| 2 Min. \| 0.24 \| 0.05 \|  \| \|  \|  \|  \| 15 Min. \| 0.27 \| 0.08 \|  \| \|  \|  \| closed eyes \| pre \| 0.60 \| 0.14 \| - \| \|  \|  \|  \| 2 Min. \| 0.58 \| 0.21 \|  \| \|  \|  \|  \| 15 Min. \| 0.60 \| 0.13 \|  \| \|  \| 1.5 T no RF (n = 19) \| open eyes \| pre \| 0.28 \| 0.08 \|  \| \|  \|  \|  \| 2 Min. \| 0.30 \| 0.06 \|  \| \|  \|  \|  \| 15 Min. \| 0.28 \| 0.06 \|  \| \|  \|  \| closed eyes \| pre \| 0.54 \| 0.09 \| pre/15 \| \|  \|  \|  \| 2 Min. \| 0.56 \| 0.12 \|  \| \|  \|  \|  \| 15 Min. \| 0.60 \| 0.11 \|  \| \| Phases one & two \| 7 T no RF (n = 31) \| open eyes \| pre \| 0.24 \| 0.06 \|  \| \|  \|  \|  \| 2 Min. \| 0.26 \| 0.07 \|  \| \|  \|  \|  \| 15 Min. \| 0.24 \| 0.06 \|  \| \|  \|  \| closed eyes \| pre \| 0.62 \| 0.17 \| pre/2, 2/15 \| \|  \|  \|  \| 2 Min. \| 0.77 \| 0.31 \|  \| \|  \|  \|  \| 15 Min. \| 0.60 \| 0.15 \|  \| |
| --- | --- | --- | --- | --- | --- | --- | --- | --- | --- | --- | --- | --- | --- | --- | --- | --- | --- | --- | --- | --- | --- | --- | --- | --- | --- | --- | --- | --- | --- | --- | --- | --- | --- | --- | --- | --- | --- | --- | --- | --- | --- | --- | --- | --- | --- | --- | --- | --- | --- | --- | --- | --- | --- | --- | --- | --- | --- | --- | --- | --- | --- | --- | --- | --- | --- | --- | --- | --- | --- | --- | --- | --- | --- | --- | --- | --- | --- | --- | --- | --- | --- | --- | --- | --- | --- | --- | --- | --- | --- | --- | --- | --- | --- | --- | --- | --- | --- | --- | --- | --- | --- | --- | --- | --- | --- | --- | --- | --- | --- | --- | --- | --- | --- | --- | --- | --- | --- | --- | --- | --- | --- | --- | --- | --- | --- | --- | --- | --- | --- | --- | --- | --- | --- | --- | --- | --- | --- | --- | --- | --- | --- | --- | --- | --- | --- | --- | --- | --- | --- | --- | --- | --- | --- | --- | --- | --- | --- | --- | --- | --- | --- | --- | --- | --- | --- | --- | --- | --- | --- | --- | --- | --- | --- | --- | --- | --- | --- | --- | --- | --- | --- | --- | --- | --- | --- | --- | --- | --- | --- | --- | --- | --- | --- | --- | --- | --- | --- | --- | --- | --- | --- | --- | --- | --- | --- | --- | --- | --- | --- | --- | --- | --- | --- | --- | --- | --- | --- | --- | --- | --- | --- | --- | --- | --- | --- | --- | --- | --- | --- | --- | --- | --- | --- | --- | --- | --- | --- | --- | --- | --- | --- | --- | --- | --- | --- | --- | --- | --- | --- | --- | --- | --- | --- | --- | --- | --- | --- | --- | --- | --- | --- | --- | --- | --- | --- | --- | --- | --- | --- | --- | --- | --- | --- | --- | --- | --- | --- | --- | --- | --- | --- | --- | --- | --- | --- | --- | --- | --- | --- | --- | --- | --- | --- | --- | --- | --- | --- | --- | --- | --- | --- | --- | --- | --- | --- | --- | --- | --- | --- | --- | --- | --- | --- | --- | --- | --- | --- | --- | --- | --- | --- | --- | --- | --- | --- | --- | --- | --- | --- | --- | --- | --- | --- | --- | --- | --- | --- | --- | --- | --- | --- | --- | --- | --- | --- | --- | --- | --- | --- | --- | --- | --- | --- | --- | --- | --- | --- |

Results of the Romberg’s test show significant changes of sway path 2 minutes after most longer lasting 7 T exposure scenarios compared to the pre-exposure measurement (“pre/2”). Shorter 7 T exposure (“7 T in & out”) and 1.5 T exposure do not generate significant changes.
