# Supplementary material for: Vestibular Effects of a 7 Tesla MRI Examination Compared to 1.5 T and 0 T in Healthy Volunteers
Source: PLoS One. 2014 Mar 21;9(3):e92104. doi: 10.1371/journal.pone.0092104 (PMC3962400; doi:10.1371/journal.pone.0092104)
Supplement: Table S2 — Unterberger stepping test results (phase 2). Results of the Unterberger's stepping test show significant changes of rotation 2 minutes after all 7 T exposure scenarios compared to the pre-exposure measurement (“pre/2”). Although only significant in the “7 T no RF & no GR” group (“2/15”), all changes are substantially but not completely reversed in the 15 minute measurement. Results after 1.5 T exposure do not change convincingly. (DOCX) [file pone.0092104.s005.docx]

| **Table S2. Unterberger stepping test results (phase 2).** | | | | |
| --- | --- | --- | --- | --- |
| Exposure group | Time point | Rotation [°] | | p < 0.05 |
|  |  | Mean | SD |  |
| 7 T no RF (n = 19) | pre | 7.8 | 18.0 | pre/2 |
|  | 2 Min. | -25.6 | 33.3 |  |
|  | 15 Min. | -8.0 | 27.0 |  |
| 7 T no RF & no GR (n = 21) | pre | 10.2 | 22.8 | pre/2, pre/15, 2/15 |
|  | 2 Min. | -21.8 | 21.1 |  |
|  | 15 Min. | -3.6 | 15.9 |  |
| 7 T in & out (n = 19) | pre | 11.1 | 28.2 | pre/2 |
|  | 2 Min. | -11.5 | 24.8 |  |
|  | 15 Min. | 2.0 | 18.2 |  |
| 1.5 T no RF (n = 21) | pre | 6.2 | 20.2 | - |
|  | 2 Min. | 3.3 | 18.7 |  |
|  | 15 Min. | 8.4 | 20.4 |  |

Results of the Unterberger’s stepping test show significant changes of rotation 2 minutes after all 7 T exposure scenarios compared to the pre-exposure measurement (“pre/2”). Although only significant in the “7 T no RF & no GR” group (“2/15”), all changes are substantially but not completely reversed in the 15 minute measurement. Results after 1.5 T exposure do not change convincingly.
